# The Rising Tide of Antimicrobial Resistance in Aquaculture: Sources, Sinks and Solutions

**DOI:** 10.3390/md15060158

**Published:** 2017-06-01

**Authors:** Joy E. M. Watts, Harold J. Schreier, Lauma Lanska, Michelle S. Hale

**Affiliations:** 1 School of Biological Sciences, University of Portsmouth, Portsmouth PO1 2DY, UK; lauma.lanska@port.ac.uk; 2 Departments of Marine Biotechnology and Biological Sciences, University of Maryland Baltimore County, Baltimore, MD 21250, USA; schreier@umbc.edu; 3 School of Earth and Environmental Sciences, University of Portsmouth, Portsmouth PO1 3QL, UK; michelle.hale@port.ac.uk

**Keywords:** aquaculture, antimicrobial resistance, fish, probiotics, horizontal gene transfer, resistome

## Abstract

As the human population increases there is an increasing reliance on aquaculture to supply a safe, reliable, and economic supply of food. Although food production is essential for a healthy population, an increasing threat to global human health is antimicrobial resistance. Extensive antibiotic resistant strains are now being detected; the spread of these strains could greatly reduce medical treatment options available and increase deaths from previously curable infections. Antibiotic resistance is widespread due in part to clinical overuse and misuse; however, the natural processes of horizontal gene transfer and mutation events that allow genetic exchange within microbial populations have been ongoing since ancient times. By their nature, aquaculture systems contain high numbers of diverse bacteria, which exist in combination with the current and past use of antibiotics, probiotics, prebiotics, and other treatment regimens—singularly or in combination. These systems have been designated as “genetic hotspots” for gene transfer. As our reliance on aquaculture grows, it is essential that we identify the sources and sinks of antimicrobial resistance, and monitor and analyse the transfer of antimicrobial resistance between the microbial community, the environment, and the farmed product, in order to better understand the implications to human and environmental health.

## 1. Introduction

### 1.1. Antimicrobial Use and Antimicrobial Resistance in Aquaculture

To feed the growing human population, global finfish and shellfish stocks (hereafter referred to collectively as ‘fish’) have been substantially exploited, with estimates of up to an 80% reduction [1,2]. To reduce this depletion, our reliance on aquaculture has intensified due to its potential to provide sustainable, safe, and reliable alternative food production systems. In 2014, 70.5 million tonnes of food fish and 26.1 million tons of aquatic algae were produced via aquaculture systems. The aquaculture production figures indicate a substantial increase in the relative contribution of aquaculture to total fish consumption from 5% in 1962, to 49% in 2002 [1]. Further estimates predict that European aquaculture production will reach 4 million tonnes by 2030 [1]. This global increase in production has also resulted in a wide diversity of species being cultivated—currently, over 580 species in total (consisting of 362 finfish and 62 crustaceans) [1,3]—with a wide range of growth and maximum production conditions.

Aquaculture systems vary in their levels of parameter control, but the main aim is to maximise the speed of growth and intensity of production, while providing safe and sustainable products [4]. To enhance fish survival and market value, a number of key strategies are regularly employed: extensive aquaculture, where predators are removed and competitors are controlled; semi-intensive, where food is supplemented and enhanced; and intensive, where all food needs are supplied. Due to the growing demands for finfish and shellfish, there has been a shift from extensive to semi-intensive and intensive systems, as they can produce greater yields [5]. However, this intensification increases stocking density and nutrient pollution, often leading to poor water quality issues. The combination of high density and poor water quality increases the likelihood of pathogen outbreaks [6], which can, in turn, have negative implications for the quality and rate of production. An outcome of these higher disease rates in intensive farming is a reliance on antibiotics and other supplements, especially in countries where regulatory limits may not be clearly defined or monitored closely [7,8].

Antimicrobial use in aquaculture is governed by a variety of factors including legislation and regulation by the respective government organization, the particular pathogen present (and its antimicrobial sensitivities), the treatment timing, the disease status of the host, and the system parameters (salinity, temperature, photoperiod, etc.). Data on the amounts of antibiotics used in aquaculture are scarce, as few countries monitor the quantity of antibiotics used [9]. However, in general, the use of antibiotics in aquaculture depends on local regulations, which vary widely. In some countries (specifically Europe, North America, and Japan), regulations on the use of antibiotics are strict and only a few antibiotics are licensed for use in aquaculture. In Europe, for example, the practice of non-therapeutic prophylactic use of antibiotics was banned in 2001 by the EU Veterinary Medicinal Products Directive, as amended and codified in Directive 2001/82/EC [10,11,12]. In Norway, stricter regulatory oversight of antimicrobial use, combined with increased vaccinations and excellent stewardship has been credited, in part, for a 99% fall in antimicrobial use between 1987 and 2013, despite output growing more than 20-fold [13,14]. However, 90% of the world aquaculture production is carried out in developing countries, which lack regulations and enforcement on the use of antibiotics [15], leading to high variability in antibiotic use. In salmon (*Salmo* spp.) aquaculture, for example, antibiotic use ranges from ~0.02–0.39 g/tonne of harvested biomass in Scotland and Norway, to ~660 g/tonne in Chile [16]. Although there is no evidence that antibiotics are routinely used as growth promoters in aquaculture, as is the case in the industrial raising of livestock in some countries [17], the prophylactic use of antibiotics in aquaculture has been commonplace in the past, particularly in shrimp [18] and salmon aquaculture [19], and the difficulty of treating individual affected finfish and shellfish means that metaphylactic use of antimicrobials to treat entire populations is still commonplace [7,15,20,21].

Antibiotics are the first line treatment for bacterial infections, and therefore play an essential role in modern medicine [22]. Antibiotic resistance is an ancient process and predates any clinical antibiotic usage [23], however, the increase of extensive drug resistant (XDR) and multidrug drug resistant (MDR) strains is a cause of immense concern. Bacteria are becoming resistant to a wide array of antibiotics as a result of natural processes [23] and widespread anthropogenic activity [24,25,26]. Bacteria can acquire antimicrobial resistance (AMR) either through mutation, or more likely horizontal gene transfer (HGT) in the environment, via natural transformation, transduction, or conjugation [27,28,29,30,31,32,33]. The genetic plasticity of the microbial community enables resistance genes to move quickly throughout different environmental bacterial populations and communities. The resistome (a collection of all AMR genes in a microbial community) in many different habitats has become an area of intense focus, with many studies examining how AMR pathways spread and evolve [34,35,36,37].

Aquaculture systems and farms have been designated as “genetic reactors” or “hotspots for AMR genes” where significant genetic exchange and recombination can occur, which can shape the evolution of future resistance profiles [38,39]. It has been estimated that 90% of bacteria originating in seawater are resistant to one or more antibiotics and up to 20% of the bacteria are resistant to at least five [40]. Once bacteria have acquired AMR genes, they may exist in the environment for a long time, even after the selection pressure ceases [41]. 

The prolonged use of antibiotics in aquaculture increases the selective pressure on bacterial populations, even at concentrations of antibiotics well below the minimum inhibitory concentration of the susceptible wild type population [42], and also increases HGT rates, including human and fish pathogens. Due to antibiotics being relatively stable and non-biodegradable, residual antibiotics can remain in commercialised fish and shellfish for consumption [7,8]. Done and Halden (2015) [43] measured low but significant levels of tetracycline (oxy- and 4-epioxytetracycline), macrolide (virginiamycin), and sulfonamide (sulfadimethoxine/ormetoprim) antibiotics in samples of farmed trout (*Oncorhynchus* spp.), tilapia (*Oreochromis* spp.), and salmon from 11 countries including the US, China, Mexico, Thailand, Scotland, and Canada. While the concentrations were in compliance with US FDA regulations, it was suggested that the presence of these antibiotics might provide a selection and enrichment mechanism for resistant bacteria [43]. Similarly, Wang et al. (2017) [44] screened finfish and shrimp samples collected from across Shanghai City for 20 common antibiotics (tetracyclines, fluoroquinolones, macrolides, β-lactams, sulfonamides, and phenicols). Antibiotic residues were found in 52% of the samples (40–91% of the finfish sampled and 17% of shrimp), with residues and their consumption accounting for 75% and 70% of the overall variance of estimated antibiotic exposure for men and women, respectively [44]. Given that 10% of the aquatic products sampled exceeded the maximum residue limits (MRL) for some antibiotics [44], it is clear that aquatic products have the potential to pose multiple health and AMR selection risks in countries where MRLs are not strictly enforced.

In addition to the use of antibiotics, other pharmaceuticals and metal-containing products are often used in aquaculture to prevent fouling, and to feed and treat fish, in order to limit the spread of infections [17]. For example, copper (Cu)-containing materials are often applied as anti-fouling agents for farm cages and nets and in the chemical control of parasites [45]; some cages themselves are made from Cu alloys [17], and high concentrations of cadmium, iron, lead [46], and mercury [47] have been reported in some commercial fish feeds. The introduction of heavy metals into the natural environment through aquaculture practices and other anthropogenic sources to the environment (e.g., the use of cadmium in pesticides and fertilizers [48]), frequently results in metal concentrations in water and sediments that exceed levels predicted to drive the co-selection of antibiotic resistance in the marine environment [49]. Therefore, the exposure of bacterial communities in and around aquaculture operations to the combination of heavy metals, antibiotics, and other co-selecting factors may further increase the likelihood of selection and co-selection of antibiotic resistance [37,38,40]. For example, previous studies on fish and eel aquaculture systems have found strains of *Aeromonas* with high levels of resistance to antibiotics and heavy-metals [50], with multiple plasmids, integrons, and gene cassettes for antibiotic resistance [51].

Fish are reservoirs of zoonotic pathogens that cannot only infect the animal host but can also infect humans who are in contact with the aquaculture facility and via foodborne infections [52]. Common infections in aquaculture facility fish handlers include *Aeromonas hydrophilia*, *Mycobacterium marinum*, *Streptococcus iniae*, *Vibrio vulnificus*, and *Photobacterium damselae* [53,54]. Although problematic as zoonotic pathogens, these microorganisms may also contain and spread AMR genes such as extended-spectrum beta-lactamases (ESBL) [5]. Faecal matter from *Sparus aurata* (Gilthead seabream) was found to contain ESBL-resistance genes, including *bla*_TEM-52_, *bla*_SHV-12_, as well as *cml*A, *tet*A, *aad*A, *sul*1, *sul*2, and *sul*3 [55]. Furthermore, bacterial strains carrying resistance determinants in commercial seafood products [56] include disease-causing pathogenic bacteria in humans [57,58], thereby increasing the risk of spreading AMR from aquaculture to the consumer. 

### 1.2. Increased Transfer of AMR Directly to the Environment through Open Systems

**Figure 1:**
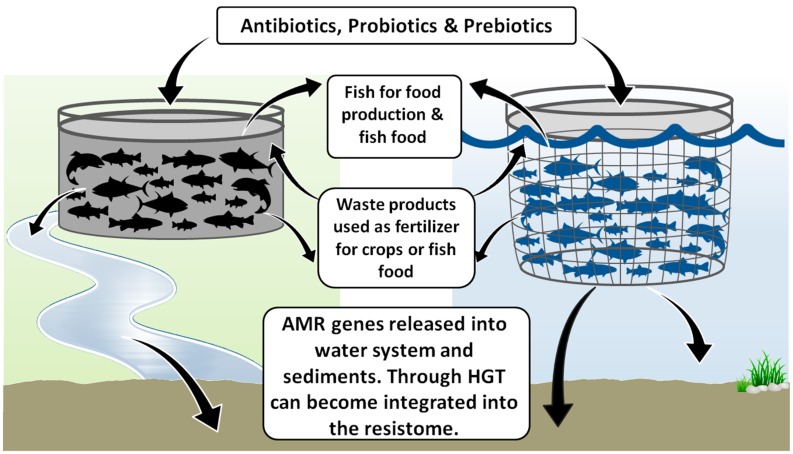
Pathways of antimicrobial resistance (AMR) genes from closed and open aquaculture systems into the water and sediment environmental resistome. See text for details.

Antimicrobial agents are usually administered to fish, mixed with food, and doses can be proportionally higher than those in livestock [13]. Not only can residues of antimicrobials remain in fish products, but undigested food and fish faeces containing unabsorbed antimicrobials and secreted antimicrobial metabolites can remain in the water and sediment around fish farms for an extensive period of time, depending on their concentrations and biodegradability [19,59]. Indeed, some studies suggest that 70–80% of antibiotics given to fish are excreted into the water [17,20,60] and can further alter the microbial communities present [61]. Such material can persist and select for AMR bacteria, even at low concentrations [42], leading to major alterations in the biodiversity of the sediment and water in the near proximity of open aquaculture systems, by replacing susceptible communities of bacteria (and other microorganisms) with resistant ones ([13] and references therein). Not only is the biodiversity altered in the surrounding sediments, but the resistome also increases in complexity, with larger numbers of antibiotic resistance genes and increases in mobile genetic elements [40,62,63,64,65]. A schematic representation of these processes is shown in Figure 1.

Sediments hold an active mix of bacterial communities, with evidence to suggest that they may be a substantial reservoir of faecal pathogens [66] and antimicrobials [67]. Detection of numerous, globally distributed, AMR genes in aquatic sediments have recently reported [32,36,68,69,70]. Various AMR genes have been documented to be present in aquatic sediment, such as *sul*1*, sul*2*, tetB, tetC, tetM, tetO, tetW, qnrA, aadA, bla_TEM_, bla_SHV_, bla_CTX-M_*, and *bla_NDM_* [59,71,72,73]. Moreover, Yang et al. [73] examined marine sediment, and found numerous tetracycline resistance genes (mentioned above). More importantly, several contigs sharing high identity with transposons or plasmids from human pathogens were detected, indicating that the sediment bacteria recently contributed or acquired resistance genes from pathogens [73]. Therefore, sediment systems are a significant environmental matrix for genetic transfer and recombination, and sediment particles offer a key interface for complex microbial community interactions, enabling AMR gene transfer [32,74].

Many studies have reported high frequencies of AMR in bacteria in the vicinity of open aquaculture operations (for reviews, see Cabello, 2006 [7]; Cabello, et al., 2013 [20]), however, few studies have been adequately designed to determine the actual impact of antimicrobial use on AMR prevalence. As Smith (2008) [9] points out, there are often issues with the media and cut-off values used for determining resistance frequencies, adequate control samples are often not included, and innate resistances are generally not considered. Other factors that may increase the frequency of AMR, such as organic enrichment, are often not considered and few studies investigate the spatial and temporal footprint of resistance. Importantly, many studies were not originally designed to investigate the linkage between the frequency of AMR bacteria and the administration of antimicrobials and do not present data on the concentrations of antimicrobials in the water and sediments. However, it is clear from a large body of laboratory and field evidence [18,59,75,76] that the use of antimicrobials in aquaculture will result in the entry of some antimicrobial compounds and their residues into the surrounding environment, which has the potential to exert selective pressure to increase the frequency of AMR in environmental microbes. For example, Zhu et al., (2017) found that concentrations of two major classes of antibiotics (tetracyclines and macrolides) were positively correlated with the total abundance of AMR genes in estuarine sediments [76].

Of particular concern are the hormetic properties of antimicrobials, where higher concentrations of antimicrobials in close proximity of aquaculture sites may select for resistant bacteria, while sub-inhibitory concentrations of antimicrobial residues in surrounding water and sediments might stimulate HGT and mutagenesis [77]. Molecular studies have shown that genes involved in AMR in bacteria associated with aquaculture are similar to those that have been detected in terrestrial bacteria associated with human and land-based animal disease [9], including in human uropathogenic *Escherichia coli* [78]. Aquatic AMR bacteria are most likely to come into contact with terrestrial bacteria and other potentially co-selective pollutants from storm-water runoff, contamination from agricultural wastes, and discharges from sewage treatment plants, in the relatively shallow coastal waters of estuaries and sheltered bays, where most open aquaculture systems are sited [79]. Tracking the flow of AMR genes is challenging, however, as gene flow may not be directly from aquatic bacteria to human pathogens, but may involve intermediaries such as other environmental bacteria or commensal microbiota of animals or humans [19]. Regardless of the pathway, the transfer of AMR genes from environmental microbes to fish, human, and animal pathogens would have a detrimental effect on piscine (both wild and farmed) and human health, and this potential link between the aquatic and terrestrial resistomes is of particular concern, as many of the antimicrobials authorised for use in farmed fish (e.g., oxytetracycline, fluorfenicol, and amoxicillin) are all medically important for human use [13]. Even when antimicrobials not associated with antimicrobial therapy in humans are selected for use in aquaculture, once the acquisition of AMR to one antimicrobial within a class occurs, cross-resistance is often conferred [80]. 

### 1.3. AMR in Closed Aquaculture Systems

Closed containment aquaculture systems refer to systems that isolate the farming process from the environment and control system parameters such as oxygenation, temperature, and photoperiods. These range from single-pass flow-through water systems to comprehensive near zero-discharge recirculating systems. As a consequence of process control and water recycling, closed systems are often seen as a sustainable practice for intensive aquaculture, dramatically reducing the amounts of waste, antibiotics, and chemical treatments reaching the environment. Closed flow-through systems produce wastewater containing suspended solids and nitrogen, phosphorous, and high microbial loads, which will either enter the municipal wastewater system after a number of treatment steps, flow to constructed wetlands, or be treated to produce a sludge that can be added to land as a fertilizer (Figure 1) [81,82,83,84]. This use of aquaculture sludge has numerous implications for the concentration and spread of AMR genes onto food crops and into the soil system [85].

Near zero-discharge recirculating aquaculture systems (RASs) are designed to produce species at high density and minimize environmental impact by effectively managing, collecting, and treating wastes that accumulate during fish growth for both freshwater and marine systems. Under optimal conditions, these systems do not require water replacement except to account for losses due to evaporation. RASs rely on both mechanical and biological filtration processes to provide an efficient, productive, and biologically secure environment [86,87,88]. Biological processes are driven by activities within microbial biofilms that develop on integrated filtration media and tank surfaces, as well as on the fish themselves, and are driven by the nutrient input. Thus, under ideal conditions, fish wastes (i.e., nitrogen in the form of excreted ammonia, and carbon and nitrogen derived from uneaten feed and faecal matter) are eliminated by the presence of interacting physiological processes including nitrification, denitrification, anammox, and methanogenesis [89]. Because system water is recycled and, under optimal conditions, very little (if any) water is exchanged with the environment, it is conceivable that antibiotics added in feed may accumulate throughout the system, promoting the growth of AMR bacteria associated with the host, the sediment (waste solids), and the RAS biofilter community. Li et al. (2017) [90] found that biofilms from RAS mixed bed biofilters are a reservoir for antibiotic resistance genes, including *tetO*, *qnrA*, and *tetE*. Biofilms, however, are generally resistant to penetration by antibiotics [90,91], which, makes the treatment of pathogens difficult [92]. While Bebak-Williams (2002) [93] found increased levels of oxytetracycline residue in sediment, biofilter, and fish muscle in a freshwater RAS after treatment with medicated feed, oxytetracycline levels decreased to nearly undetectable levels with time after withdrawal of the drug. Very little is known about the occurrence of AMR bacteria in RASs and those that have screened for AMR pathogens concluded that their presence could be explained by the use of infected fish stocks [94,95]. Table 1 details some of the representative antimicrobial resistance factors that have been detected in aquaculture facilities across the world.

### 1.4. Integrated Fish Farming and “Waste as Feed”

In addition to the direct therapeutic application of antibiotics to aquaculture systems, integrated fish farming is another potential source for antimicrobial residues and AMR bacteria in aquatic environments in and around aquaculture farms. Integrated fish farming is a finfish or shellfish production system that combines other agricultural/livestock farming operations and is practiced widely throughout Asia and Africa. Finfish or shellfish are typically raised in ponds with units of livestock, such as pigs, cattle, and poultry, located over or near a pond, allowing drainage of livestock manure and excess feed into the pond as direct feed for fish and/or as fertiliser for phytoplankton and other live fish feed [103]. The livestock are usually reared intensively, with antimicrobials used as growth promoters and for prophylactic and therapeutic treatment [104]. Therefore, although such systems are considered sustainable in many ways, they also pose potential food safety hazards including transmission of AMR bacteria and faecal zoonotic pathogens, as well as accumulation of antimicrobials and other chemical residues [105].

Very few studies have investigated the relationship between antibiotic contamination and AMR in aquatic environments relating to human and agricultural activities within integrated farming systems. Peterson et al. [80] studied integrated chicken-fish farms in Thailand and found a significant increase over time in the resistance of *Acinetobacter* spp. to six different antimicrobials, with resistance to oxytetracycline and sulfamethoxazole increasing from between 1% and 5% to 100% within 2 months of a new fish production cycle. Of great concern was that when looking at the long-term effects of integrated farming, AMR genes were particularly prevalent among *Enterococcus* spp. [80], some of which are known to cause clinical infections in humans. Selection for AMR appeared to occur in the gut of the chicken, with AMR higher in the chicken manure than for isolates from water-sediment samples. Upon release into ponds, AMR bacteria from livestock manure may act as donors of AMR genes, or their presence may be favoured due to selection pressure exerted by the presence of antimicrobials or antimicrobial residues. Other studies have also shown that integrated agriculture/aquaculture systems are reservoirs for antimicrobials and AMR genes [14,104,105]. Because ponds function as water storage systems and are not subject to frequent exchange, AMR bacteria and antimicrobials accumulate in pond water and sediments and there is adequate time for bacteria to develop resistance by promoting HGT [106]. During water exchange and at harvest time, pond water is often released as a point source into rivers, estuaries, or the sea [107], potentially disseminating AMR bacteria and antimicrobials into the wider aquatic environment.

Recovery of fish-processing wastes for aquafeeds (“waste as feed”) has also been suggested as a means of increasing the sustainability of aquaculture, by reducing dependence on wild fisheries for fishmeal and fish oil. Currently, between 30% and 70% of the volume of fish biomass ends up as wastes, depending on the type of fish processing level [108], so recycling waste as feed is an attractive prospect. However, the use of fish-processing wastes, particularly those originating from aquaculture, raises additional concerns about the transmission of AMR bacteria, as well as concerns about the bioaccumulation of contaminants (including antimicrobials), and cross-species transmission of pathogens. Although few studies have examined the effects of integrated fish farming or “waste as feed” approaches on the occurrence of AMR, the findings of these studies raise great concerns regarding the long term effects of antibiotic use in agriculture and aquaculture systems, and it is clear that these methods of farming pose substantial risks to human and environmental health.

### 1.5. Probiotic Application

Probiotics were initially described by Parker (1974) [109] as “microbes or substances that improve intestinal balance of a host animal”. However, a Food and Agriculture (FAO) and World Health Organisation (WHO) joint report (2001) [110] refined the definition as “live microorganisms which, when administered in adequate amounts, confer a health benefit on the host” [111]. This change in definition reflects the increase in understanding of the complexity of the host microbiota interactions. Kozasa (1986) [112] reported the first application of probiotics in aquaculture, using *Bacillus toyoi* spores as an additive in feed for yellowtail. Aquaculture probiotics can be acquired from a wide range of sources—host or non-host derived cultures may be used; host derived probiotics are generally isolated from fish digestive tracts, skin mucus, and gills, suggesting that they are part of the resident microbiota, as well as from system water and component surfaces (associated with biofilms) [113]. Probiotics generally refers to bacteria—both Gram-positive and Gram-negative members of diverse phyla (for reviews see Adel et al. [114] and Pérez-Sánchez et al. [115])—and fungi (e.g., *Aspergillus* sp. [116] and *Saccharomyces* spp. [115,116]), however, microalgae (e.g., *Tetraselmis suecica*, which is inhibitory to *Aeromonas* and *Vibrio* spp.) have also been shown to possess probiotic characteristics [117]. The regimens and application methods can have a number of effects on AMR transfer within aquaculture. The high doses of bacteria often added daily, could cause major shifts in the microbial community present, possibly leading to an excess of AMR species within the aquaculture system.

The antibacterial effect exhibited by probiotics is due to a variety of factors including the production of antibiotics, iron-scavenging siderophores, enzymes (e.g., proteases, amylases, and lysozyme), hydrogen peroxide, organic acids (which in mammals may alter the host’s intestinal pH), and bacteriocins [118]. Bacteriocins are proteinaceous toxins produced by a wide range of bacteria and archaea and have a number of properties similar to antimicrobials that make them ideal candidates for pathogen control: potency, mode of action, target cells receptors, size, and structure [119]. Although their high selectivity make bacteriocins attractive alternatives to classical antibiotics, initial studies indicate that bacteriocinogenic bacteria may harbour AMR genes [119]. Specific AMR determinants carried on mobile genetic elements constitute a reservoir of resistance for potential food or gut pathogens, thus representing a serious safety issue [120] and highlighting the importance of assessing AMR susceptibility when prospecting bacteria for use as probiotics. 

Probiotics may offer alternatives to antimicrobial compounds, however, microbes used as probiotics are not exempt from acquiring antibiotic resistance genes via HGT. Given their shared microbial environment in the gastrointestinal tract, there is a risk of probiotic microbes acquiring antibiotic resistance genes from pathogenic microbes, and vice versa [120]; Munoz-Atienz (2013) reported the presence of several antibiotic resistance genes in lactic acid bacteria of aquatic animal origin that were intended for use as probiotics in aquaculture [121]. The long term implications of adding high numbers of live bacterial populations to aquaculture systems that may still have high levels of AMR genes and antimicrobials in situ, needs to be further examined. 

## 2. Conclusions and Perspectives

The persistence and proliferation of AMR in the environment represents a global health crisis, with a current estimate of 700,000 AMR deaths attributed annually and estimated to rise to 10 million deaths per year in 2050 [121]. Furthermore, by 2050 AMR could cost $100 trillion in lost economic output [122]. Therefore, it is critical that we better understand environmental hotspots for genetic exchange of AMR genes such as aquaculture systems and determine how they might transfer to clinically relevant strains. 

In this review we have highlighted a number of critical areas that facilitate and promote antimicrobial gene transfer in aquaculture systems. Some essential areas for further study include: the role of probiotic microorganisms in HGT of antibiotic resistance genes; the ability of AMR genes to transfer to the host organism; the role of waste when used as a food or a fertilizer as a potential source of AMR genes and issues with cross species AMR factor interactions; and the resilience of AMR genes in aquaculture systems. Globally, wide ranges of products are farmed in many types of aquaculture systems operating under the control of a myriad of legislative policies. A consistent approach to AMR control and food safety is required in order to reduce the threat of worldwide resistance. Although considerable studies have been performed in other intensive food production areas such as pig and poultry farming, there is currently a lack of extensive studies in aquaculture systems. International efforts to better understand the transfer and stability of AMR are required to fully understand these mechanisms and develop strategies for their mitigation.

Since the use of antibiotics for disease inhibition and as growth promoters have been prohibited in Europe and regulated in other countries, alternative strategies have been used to alleviate pathogen activity including: vaccination [123]; immune stimulation using nutritional factors derived from bacterial, algal, or animal (including hormones and cytokines) sources [124]; phage therapy [125]; and quorum sensing disruption (affecting virulence) [126]. In addition, the disinfection of system water may be managed with UV application [127] or, as is often the case for intensive systems, via ozone treatment [127,128]. These alternative strategies combined with a better understanding of the effects on the microbiome of the farmed host may provide alternative solutions to improve aquaculture health and function, while reducing the potential for the spread of antimicrobial resistance. 

## Figures and Tables

**Table 1:** Representative microbial antibiotic resistance determinants identified from aquaculture systems.

Antibiotic Class	Antibiotic Resistance Gene	Aquaculture System or Fish Species	Reference
β-Lactam (e.g., Ampicillin, Amoxicillin)	*bla*_TEM-52_, *bla*_SHV-12_	Gilthead Seabream	Sousa et al. [55]
	*bla*_TEM_	Fish farms, Pakistan and Tanzania	Shah et al. [96]
Tetracycline (tetracycline, oxytetracycline, chlortetracycline)	*tetM, tetO, tetT, tetQ*	Fish farms, Tianjin, and Guangdong, China	Gao et al. [64], Xiong et al. [65]
	*tetM*, *tetS*	Japanese and Korean coastal farms	Kim et al. [97]
	*tetA, tetG*	Chilean salmon	Shah et al. [98]
		Fish farms, Pakistan and Tanzania	Shah et al. [96]
	*tetA*	Marine aquaculture, Spain and Portugal	Rodriguez-Blanco et al. [99]
	*tetA, tetB, tetK*	Salmon aquaculture, Chile	Buschmann et al. [19]
Sulfonamide, sulfamethizole	*sul1*, *sul2*, *sul3*	Fish farms, Tianjin, China; farmed freshwater fish, Guangdong, China; Gilthead seabream	Sousa et al. [55], Gao et al. [64], Xiong et al. [65]
	*sul1*, *sul2*	Chilean salmon; fish farms, Tanzania and Pakistan	Shah et al. [96], Shah et al. [98]
Aminoglycoside (Streptomycin, spectinomycin, neomycin)	*aadA strA-strB*	Chilean salmon, fish farms, Tanzania and Pakistan; catfish farm, Vietnam; carp farms, Poland	Shah et al. [96], Shah et al. [98], Nguyen et al. [100], Piotrowska et al. [101]
	*aad1*	Gilthead Seabream	Sousa et al. [55]
Amphenicol (chloramphenicol, florfenicol)	*cmlA*	Gilthead Seabream	Sousa et al. [55]
	*cat-1*	Fish farms, Tanzania and Pakistan	Shah et al. [96]
	*floR*	Salmon aquaculture, Chile	Buschmann et al. [19]
	*catB*	Catfish farm, Vietnam	Nguyen, et al. [100]
Quinolones (oxolinic acid, ciproflaxin)	*qepA*, *oqxAB*, *qnrS*, *aac(6′)-Ib, qnrB, qnrD*	Farmed freshwater fish, Guangdong, China	Shah et al. [98], Jiang et al. [102]
	*qnrA, qnrB, qnrS*	Salmon aquaculture, Chile	Buschmann et al. [19]
Macrolides (erythromycin)	*mefA*	Fish farms, Tanzania	Shah et al. [96]
	*ermC, ermE, ermX, ermC*	Carp farms, Poland	Piotrowska et al. [101]
Trimethoprim	*dfrA1*, *dfrA5*, *dftA12*	Chilean salmon; fish farms, Tanzania and Pakistan	Shah et al. [96], Shah et al. [98]
	*dfrA12*	Catfish farm, Vietnam	Nguyen et al. [100]
Quinoxoline 1, 4-di-*N*-oxides (carbadox, olaquindox, mequindox)	*oqxA*	Salmon aquaculture, Chile	Buschmann et al. [19]
